# Mr. Dennett, on Cow-Pox

**Published:** 1801-09

**Authors:** Christ Dennett

**Affiliations:** Surgeon. Soho Square


### *To the Editors of the Medical and Phyfical Journal.*

GENTLEMEN,

IF the inclofed cafe, and obfervations thereon, fhall be found to contain information deferving a place in the Medical and Phyfical Journal, it is much at your fervice, and the infertion will greatly oblige,

Yours, &c.

CHRIST. DENNETT, Surgeon.

*Soho Square, July* 28, 1801.

---

IN the prefent ftate of Vaccine Inoculation, every cafe which deviates in any degree from the regular progrefs, either under particular circumftances or in appearances, ought to be noticed. The following, in my opinion, is one of thefe.

On the 9th of November laft I was called to vifit the child of Mr. P——d, who I found had got the natural Small-pox of the diftinct kind, which had made its appearance on the evening of the 7th. As there was a brother who had not had the diforder, I thought this would be a proper trial of the preventive effect of the Cow-pox; accordingly I immediately inoculated him in the left arm with Vaccine matter I had by me, taken on the 4th of the month.— The child was about twelve months old. On the 11th, there was a flight degree of inflammation, but very pale. On the 12th, it had quite difappeared. I this day inoculated him again in the right arm, with recent virus, taken half an hour before; this had the defired effect. On the 15th, it looked red, and might be diftinctly felt, but no inflammation attended the firft incifions. On the 17th, the right arm looked very florid; there was not the flighteft appearance of inflammation on the left, but the incifions might be feen. I was informed that the child had two puftules in its face; on examination, it appeared to me only a violent humour from teething, for the furface was flat and as large as a filver penny, attended with feveral inflamed pimples about the fize of a fmall pin's head. The left parotid gland was alfo much enlarged, which the mother attributed to cold. On the 18th, the right arm was inflamed more than the day before, but I did not examine the left. On the 19th, I underftood the child had paffed a very reftlefs night, and found he had then confiderable fever. The inflammation on the right arm extended as large as a half crown. The mother greatly furprifed me when fhe

ſaid that the left arm had now began to inflame a little; this I found to be the caſe, attended with a ſmall puſtule in the centre of the inciſions, reſembling the Small-pox. I this day took matter from the right arm for preſerving. 20th, The child had paſſed a better night, but ſtill feveriſh; the inflammation going off both arms; the humour in the face much the ſame. I this day took matter (on a lancet) for a friend, from the right arm, but it failed. I afterwards found he had inoculated the ſame child twice before, without effect. On the 21ſt, the febrile ſymptoms nearly ſubſided, which have been much more ſevere than in any patient I have before inoculated with the Vaccine virus. On the 22d, the inflammation nearly gone; the veſicle on the right arm getting dry; but on the left the puſtule increaſing; fever quite gone. On the 23d, the veſicle on the right arm formed into a dry brown cruſt; the puſtule on the left had every appearance of being variolous, of which I informed my aſſiſtant, and requeſted that he would viſit the child in the afternoon, to obſerve the ſimilarity; and at the ſame time take matter from the puſtule. He did not think it ſufficiently mature, however he took a little. The ſpots on the face were dry, and had much the appearance of the right arm, except being more flat. On the 24th, I took matter from the puſtule on the left arm. From this day the child required no further attendance.

With the matter taken from this child I have continued, in ſucceſſion, inoculating patients to the preſent period. That taken from the right arm has produced, in all, the true Vaccine diſorder, in its moſt mild form; and that taken from the left produces the Variolous.

This caſe, with many others, particularly thoſe attended with eruption, inoculated by Dr. Woodville at the Small-pox Hoſpital, and the one ſtated by Mr. Malim, of Carey Street, is deciſive, that expoſure to variolous contagion at the time of the inoculation for the Cow-pox is highly improper, and ſhould be a caution to every practitioner, when he has reaſon to ſuppoſe the patient has been expoſed to variolous effluvia; for although only local inflammation and veſicle be produced from ſuch inoculation, (as in this caſe) yet much diſcredit may be brought againſt the practice, if great circumſpection is not uſed: but, at the ſame time, I think no one who has been much accuſtomed to the Small-pox, can be deceived in the appearance.—This child, I conclude, received the infection from his brother during the eruptive fever, which counteracted the firſt inoculation ſo far as only to produce a ſimple puſtule.

As it muſt be a deſirable conſideration to the profeſſion at large to be in poſſeſſion of a method for keeping the Vaccine matter matter, fo that it may not fail, I fhall beg to lay before the numerous Readers of the Medical and Phyfical Journal the plan I have found fucceed beft, and the manner of inoculation. I have practifed the fame in the inoculation for the Small-pox twenty years, (having had every opportunity for obfervation, while living with my father, whofe extenfive practice has been well known throughout the county of Suffex upwards of forty years) and it has fcarcely ever been found to fail; indeed fuch fuccefs has attended it, that I was about communicating the manner of my treatment to the public; but when I found the Vaccine inoculation fo generally received, I declined it, and fhall now wait the event of further experience in the Cow-pox inoculation; for there are few of my patients who will permit me to practice it as I could wifh.

When I intend to preferve the Vaccine virus, I ufually take it on the eighth day, and puncture the veficle in two or three places with the point of a lancet, then wait half a minute or a minute until the pellucid matter oozes out, which I take on the end of a quill, *and on the convex fide*, cut in the manner of a tooth-pick, but not quite fo tapering, with the point fomewhat rounded, and a little bent, to prevent the matter being rubbed off in paffing it into another quill, which I cut ftrait at the larger end, taking care to have them fit well before I attempt to take the matter. In this manner I have kept Variolous matter good for years; and have ufed Vaccine, thus preferved, four, five, fix, and in one inftance near feven months, yet have never found it fail, nor attended with too violent inflammation, or other bad fymptom.—I have now matter by me taken in November laft, which, I doubt not, would produce the Vaccine diforder. In this ftate it may be fent to any part of the world with certainty of fuccefs. The manner of my inferting it is by making three or four incifions or fcratches acrofs the arm, (as defcribed by Dr. Paterfon, in the laft number of the Medical and Phyfical Journal) about one-fifth of an inch long, and half a line apart: thefe ought to be made in the flighteft manner poffible, as no more blood fhould be produced than what may ferve to moiften the matter, which is to be repeatedly rubbed over the incifions until all be diffolved. If it has not been taken more than a day or two, it will require very little trouble to infert; but if it has been kept any length of time, I then hold it over the vapour of hot water for half a minute or a minute.—The quills ufually employed for pens I find too hard, therefore felect what are called the large feathers.

